# Technocratic Decision-Making in Times of Crisis? The Use of Data for Scientific Policy Advice in Germany’s COVID-19 Management

**DOI:** 10.1007/s11115-022-00635-8

**Published:** 2022-06-10

**Authors:** Sabine Kuhlmann, Jochen Franzke, Benoît Paul Dumas

**Affiliations:** Faculty of Economics and Social Sciences, University of Potsdam, August-Bebel-Straße 89, Building 7, 14482 Potsdam, Germany

**Keywords:** Data utilization, Scientific policy advice, Data culture, Data literacy, COVID-19 crisis

## Abstract

COVID-19 has demonstrated the importance of data for scientific policy advice. Mechanisms by which data is generated, shared, and ultimately lead to policy responses are crucial for enhancing transparency and legitimacy of decisions. At the same time, the volume, complexity and volatility of data are growing. Against this background, mechanisms, actors, and problems of data-driven scientific policy advice are analysed. The study reveals role conflicts, ambiguities, and tensions in the interaction between scientific advisors and policy-makers. The assumption of a technocratic model, promoted by well-established structures and functioning processes of data-driven government, cannot be confirmed. Reality largely corresponds to the pragmatic model, in parts also the decisionist model, albeit with dysfunctional characteristics.

## Introduction

The utilization of data for policy advice has become an increasingly salient feature of politico-administrative decision-making in recent years (Christensen, 2021; Duina, 2021; Zarkin, 2021). The mechanisms by which data is generated, shared, and ultimately lead to evidence-based policy responses are crucial for enhancing the transparency and legitimacy of decisions, particularly in situations of crisis, such as the COVID-19 pandemic (Pattyn et al., 2019; Sager et al., 2020). At the same time, the volume, complexity and volatility of the available data are also growing (Kayser & Blind, 2017). This imposes additional pressures on policy-makers, who seek to use data-driven policy advice to reduce ambiguity and uncertainty (Sell et al., 2021). Actors involved in scientific policy advice are also facing these challenges because they are expected to offer accurate and implementable advice, often based on rapidly evolving, sometimes uncertain and even controversial data that are available at the moment. Furthermore, decision-making processes are not simply guided by principles of evidence, information and knowledge, but also follow the logic of power-seeking, consensus-building, compromise and political feasibility (Sager et al., 2020). Consequently, the question arises, how to organize institutional processes in a way that the logic of evidence and knowledge could be strengthened without neglecting the logic of politics or even drifting off towards a technocratic predominance of scientists.

During the COVID-19 pandemic, these tensions have become particularly evident. The crisis has not only demonstrated the crucial role of data for scientific policy advice and decision-making (data was referred to as the “staff of life” - “*das tägliche Brot*”; Interview 10, see Appendix Table 1). It has also revealed some glaring deficiencies and limitations regarding the existing advisory settings, digital maturity of processes and institutional mechanisms of data generation, sharing, and usage (Weingart, 2021). Against this background, this article analyses the existing mechanisms, actors, modes and problems of data-driven scientific policy advice during the COVID-19 crisis in Germany, providing an empirical overview of the current processes and highlight the key challenges of data generation and utilization. The analysis will shed light on the dynamics and conflictual interplay between data users in politics and public administration and scientific data providers at all levels of the German federal system. The objective is to identify the strengths and weaknesses of digital data generation, transfer, and utilization for crisis management and to identify potential areas for improvement in order to be better prepared for future crises.

The following research questions will be addressed:

- How did scientific data providers and politico-administrative data users interact during the COVID-19 crisis?
- Which gaps and deficits have become apparent concerning data availability and accessibility as well as digital data sharing and transfer?
- How was (digital) data integrated with politico-administrative processes of decision-making?

Our analytical framework consists of two major dimensions: (1) the interface and operational rationalities of science, public administration and politics (interface-models); (2) the institutional contexts of data utilization (context). Regarding the first dimension, which represents our dependent variable of analysis, we make a distinction between the technocratic, decisionist, and pragmatic model of policy advice, and ask to what extent the reality of data-based scientific policy advice during the COVID-19 crisis resembles one or more of these models. With respect to the second dimension (explanatory variables) we concentrate on the aspects of digital maturity, data culture, and data literacy in public administration and examine their influence on data-based scientific policy advice and political decision-making.

Methodologically, the present study is based on 16 semi-standardised interviews. The semi-standardised character of the interviews aims at authentic data on experiences of particular events. Thus, the interviews allow for individual emphasizing by the interviewees without pushing them through the structure of the interview (Crouch & MacKenzie, 2006: 485ff). As interviewees, experts at all levels of government were chosen, including political, executive and administrative actors as well as internal and external policy advisors from various disciplines and institutions.^1^ In accordance with Diefenbach, the selection of interviewees was emphasized (Diefenbach, 2008: 879). In this context, it was especially important to include actors representing the political executives, such as heads of municipalities (Interview 3) as well as heads of department and top-level bureaucrats being able to explain the perspective of the respective ministers and secretaries (Interviews 10, 11). Furthermore, an analysis of the current state of the academic and public debate was taken into account, including relevant documents related to COVID-19 management and data strategies of all governmental levels in Germany, media coverage and other available reports as well as so-called ‘grey literature’ on the topic under investigation.

The analysis will proceed as follows: First, some background information about German pandemic crisis management will be provided (“Background: Basic Features of Pandemic Crisis Management in Germany” section) and our study’s concept will be introduced (“Conceptual Framework: Interface-models and Contexts” section). Second, the empirical findings of data-based scientific policy advice in Germany referring to data gaps and deficits will be presented (“Data Gaps and Deficits”) as well as the interface between science, public administration and politics (“The Interface Between Science, Public Administration and Politics”), and the institutional contexts of data utilization (“Institutional Contexts of Data Utilisation”). In the discussion section we will apply our conceptual framework on the empirical data (“Discussion: The Role of Interfaces and Contexts for Data-Based Scientific Policy Advice” section). Finally, a future outlook will be provided (“Conclusions” section).

## Background: Basic Features of Pandemic Crisis Management in Germany

Germany’s specific intergovernmental set-up as a ‘unitary federation’ (*unitarischer Bundesstaat*) with 16 federal states (*Länder*) and functionally strong local governments that enjoy a powerful position and great autonomy (see Kuhlmann & Wollmann, 2019) must be taken into account when it comes to analyse pandemic management in this country (for details see Kuhlmann & Franzke, 2021). Within this highly decentralized and fragmented institutional setting, the unity of law, economy and living conditions are however constitutionally protected. Multiple mechanisms are provided for the enforcement of collaboration and joint decision-making across levels and jurisdictions in order to guarantee the unity of the federation (see Behnke & Kropp, 2021; Kuhlmann & Franzke, 2021).

During the pandemic, the federal government did not make use of the constitutional emergency regulations for defence or natural disasters, but based crisis management mainly on the Federal Law on the Protection against Infections (*Infektionsschutzgesetz* – IfSG), which stipulates an exclusive administrative competence of the *Länder* and local governments. According to the general clause (§ 28) of the IfSG, the *Länder* and the local governments are exclusively empowered to take containment measures, whereas the federal government is bound to recommendations and incentives. Within this traditional legal framework of epidemic mitigation, the *Länder* and local governments can enact executive orders to temporarily impose lockdowns, contact-bans, shutdowns and closures of public facilities and thus suspend fundamental civil rights – limited in time and space. Direct interventions of the federal government are meant to be limited in the event of an epidemic outbreak.

Yet, this setting changed quite significantly over the course of the pandemic with the result of regulatory powers of the federal government being strengthened considerably. The Federal Ministry of Health, in particular, gained (temporary) additional means to issue ordinances, even without the consent of the *Länder* and the *Bundestag*. The introduction of the so-called “federal emergency brake” in April 2021^2^ can be regarded as a preliminary climax of pandemic-related standardization and centralization, since, for the first time in the pandemic, a centrally imposed rule applied, which remained in force until the end of June 2021 when the law expired and Germany returned to the more decentralized approach of crisis management.

The predominance of sub-national actors in German pandemic management does not lead to disconnected and completely discretionary action – quite on the contrary. In line with the principles of a “unitary” and “cooperative federalism” (see above), intense coordination and collaboration across levels and jurisdictions were extensively practiced during the pandemic. Throughout the crisis, phases of intense coordination and “unitarization” of decision-making, especially when the pandemic situation was perceived as aggravating, alternated with phases of looser intergovernmental collaboration and more discretionary regulatory powers of the *Länder* and local governments, particularly when the situation was perceived as more relaxed and lifting of measures as justifiable (see also Schnabel & Hegele, 2021). Regulatory unitarization and negotiated alignment of measures were predominant in phases of perceived crisis aggravation whereas in times of perceived relief, subnational discretion and more regional variance of containment rules appeared to be appropriate. As a result, the regulatory landscape (lockdown rules etc.) looked quite homogeneous in different German regions, some criticism about an alleged federal mess in the *Länder*-specific details of containment notwithstanding.^3^

## Conceptual Framework: Interface-Models and Contexts

In the context of knowledge utilization in general, scientific policy advice represents a specific type of knowledge due to its decision-oriented character and due to the requirement of being “fit for function” (Funtowicz, 2001). However, the provision of scientific knowledge for political decisions implies significant difficulties (Falk et al., 2006: 13). Inter alia, such problems arise from different operational rationalities of knowledge producers and principals. Scientific policy advice is expected to fulfil the double function of pushing evidence-based policymaking while improving the legitimacy of political decisions (Bogumil, 2018: 157). Scientific policy advice thus aims to produce knowledge that is not only “factually correct and resilient”, but also “politically useful and utilizable” (Weingart & Lentsch, 2008: 17, 53ff.).

Drawing on Esty and Rushing (2007), data-driven policy advice is conceived as the collection, analysis and utilization of data with the aim of understanding, explaining and resolving crisis-related policy issues and to further propose potential solutions to policy-makers (ibid.: 1). When explaining data utilization for scientific policy advice in the COVID-19 pandemic, we make an analytical distinction between two key dimensions: on the one hand, the (rival) operational rationalities and the dynamic interplay between science, public administration and politics must be taken into account. On the other hand, we focus on the institutional contexts of data utilization.

### Interface-Models and Operational Rationalities of Science, Public Administration and Politics

Regarding the above-mentioned conflicting rationalities, scholars distinguished the “two worlds” of scientific policy advice on the one hand and politico-administrative decision-making on the other (Habermas, 1969).^4^ The academic debate evolved around the peculiarities, specific logics, yet also increasingly the interface, boundaries and boundary-spans between these worlds. In the so-called “decisionist model”, politico-administrative actors are conceived as dominating the process and making use of experts’ knowledge in a purely instrumental way, assuming that this knowledge is value-free. The “technocratic model”, by contrast, assumes a superiority of scientific expertise based on the belief that science can identify optimal solutions to every problem, thus reducing politico-administrative actors to mere executors of scientific directives (Böcher, 2007; Martinsen & Rehfeld, 2006; Schelsky, 1979; Weber, 1988). These ideal types, at best partially representing reality, have been contrasted with the so-called “pragmatic model” as a third, more realistic model. Accordingly, politico-administrative decisions are seen as results of an iterative process between actors actively involved either in scientific policy advice or in decision-making. This implies an ongoing “translation process between science and politics” (Habermas, 1969: 137). Metaphors such as “boundary spanning” (see e. g. Martinsen & Rehfeld, 2006: 48) have been used to describe this particular process. More recent concepts, such as the recursive model of scientific policy advice (Weingart, 2001) or the concept of “boundary work” (Korinek & Veit, 2013), similarly emphasise the complex interdependent relationships and negotiation processes between science and politics.

The concept of “boundary work” builds on assumptions such as the above-mentioned importance of knowledge as a resource of bureaucratic power postulated by Weber (2006: 226). Also linked to Habermas’ pragmatic model of scientific policy advice, the concept assumes that both politics and science aspire for expansion into each other’s area of competence. Consequently, the respective boundaries between science and politics are subject to constant negotiations, thus resulting in scientific policy advice being an essential part of a “boundary world” (Gieryn, 1983: 791f; Korinek & Veit, 2013: 267, Sokolovska et al., 2019). As a result of this, scientific policy advice is to be characterised by a high degree of uncertainty of respective expectations. This structural feature of scientific policy advice can significantly complicate data-driven policies (Gerlinger, 2019; Haucap, 2020; Weingart, 2019). Therefore, the involvement of ‘brokers’ who provide the necessary ‘translation services’ is required.

Illustrating the need for such brokers, it is to be stressed that scientific policy advice often relies on contextualization and nuanced interpretations, whereas policy-makers expect unambiguous interpretations, clear conclusions as well as recommendations for practical implementation or even implement trial-and-error strategies (Boin & Lodge, 2021). Further complication arises from the fact that policy-makers tend to normatively contextualize scientific data in order to allow for necessary considerations with other normative criteria based on societal expectations (Gerlinger, 2019). Hence, “the same data (can) also lead to several translations of different sense”, so that the “ambiguity of data (…) opens up the possibility of different interpretations” (Schwab, 2014). Correspondingly, the need for such negotiation processes in the context of data utilization “challenges the rational decision-making model of performance-oriented reform concepts” (ibid.). Various authors (e.g., Moynihan, 2008, cited in Schwab, 2014) therefore propose procedures of discursive and interpretive processing of data or refer to the context-dependency of data utilization.

### Institutional Contexts of Data Utilization

To explain data utilization in crisis-related advisory and decision-making processes, institutional contexts must be taken into account. It is assumed that varying degrees and scopes of data utilization in organizations are shaped by formal organizational features and regulations on the one hand and administrative cultures and informal rules on the other (see Christensen & Lægreid, 2020; Kuhlmann et al., 2021). Whereas formal regulations (e.g., privacy rules) and structures (e.g. organizational hierarchies, digital maturity of processes) shape the macro-setting of data-based advice and limit corridors of action, informal norms (e.g., data culture, data literacy) and identities generate a “logic of appropriateness” (March & Olsen, 1989) which impacts on the meso- and micro-levels of organizations. Against this background, e-government research has identified process-related, technological as well as legal hurdles of data utilization (Kempeneer, 2021; Matheus et al., 2020). Different types of data strategies (e.g., internal and external orientation, cf. Van Donge et al., 2020) may determine data utilization, as they institutionalize the data culture of the respective institutions. In this context, the digital maturity of public organizations (see Kuhlmann & Heuberger, 2021) represents a key variable to explain data utilization in advisory settings. The varying levels of progress in the digital transformation of public administration thus appear to be relevant explanatory factors for the extent and intensity of ICT-based data utilisation in crisis-related policy advice. Of course, the relationship between data utilization on the one hand and institutional contexts on the other hand also exists vice-versa: institutions may not only shape data utilization, as the latter might also shape the respective institution (Christensen & Laegreid, 2020: 4).

### Hypotheses on the Influence of Context Conditions on Data-Based Policy Advice

In our analytical framework, the interface models (technocratic, decisionist, pragmatic) are conceived as dependent variables of investigation, while the context conditions (data management, digitalization, data culture) are considered as explanatory variables. We assume that the institutional context influences the mode of policy advice and decision-making. More precisely, favourable conditions for data-based scientific policy advice are expected to promote technocratic policy-making, as investments in such context conditions suggest a demand for data-driven and therefore rather technocratic policy-making. This refers inter alia to Germany’s comprehensive efforts towards the digitalization of public administration and the accompanying financial support to speed up the progress of digital transformation in the public (health) sector (see Kuhlmann & Heuberger, 2021). Furthermore, the public health service with its well-established and internationally renowned surveillance and reporting system, as well as Germany’s position as a frontrunner in health-related research (e.g., in virology, epidemiology) suggest a favourable context for data-based scientific advice and policy-making. Because of the particularities of the COVID-19 crisis, especially the importance of data as a strategic key resource in decision-making, we expect these conditions to facilitate a predominantly technocratic model of policy advice in which scientific advisors and data-providers are privileged vis-à-vis to policy-makers. Respective expectations are backed up by first insights from public administration literature in Germany, underlining the hypothesis of a predominantly technocratic mode of policy advice and policy-making in Germany (cf. Böcher et al., 2021). Going one step further, Florack et al. postulates a “Coronacracy” (Florack et al., 2021). In addition, the uncertainty and volatility of data and knowledge especially at the beginning of the crisis pushes the demand for scientific policy advice in order to legitimize political decisions in such contexts, thus paving the ground for a technocratic dominance in the policy-making process.

## Data-Based Scientific Policy Advice during the COVID-19 Crisis: Empirical Findings

According to the theoretical approaches mentioned above, data-based scientific policy advice and decision-making are influenced by two essential elements: first, the operational rationalities and the interface between science, public administration and politics. Second, the institutional contexts which shape data generation, transfer as well as the type, scope and quality of the data presented to policy makers. Before exploring these two elements in greater detail, we will first outline major data gaps and deficits which have been revealed in the COVID-19 crisis management.

### Data Gaps and Deficits

Irrespective of different rationalities, data-based scientific policy advice and decision-making are foremost dependent on the availability and accessibility of scientific data. However, the COVID-19 crisis has demonstrated that these requirements have not been met sufficiently. Today, there are still significant data gaps that impede evidence-based advice as well as decision-making. Despite of complements to existing surveillance and health monitoring systems initially established for non-crisis periods, considerable quality problems and data deficiencies have come to light.

#### Epidemiological and Capacity-Related Data Gaps

Initially, data-based scientific policy advice and data utilization by political-administrative decision-makers were focused on virological and epidemiological data (such as the RKI^5^ dashboard and local health department data) as well as on resource- and capacity-related health care data (such as the DIVI register,^6^ local hospital data). The demand for data estimating indirect and consequential effects of the measures only emerged as the crisis progressed, aiming for a more holistic pandemic assessment especially including economic and societal effects of the pandemic management (see Schmidt, 2020).

Regarding epidemiological data, criticism concerns a lack of knowledge on the socio-economic backgrounds of patients hospitalised in intensive care units. Additionally, a lack of representative cohort studies generating systematic data on the hazards of COVID-19 as well as on risks of infection and death was criticised. Respective studies were requested to include systematic information on age groups, previous illnesses, regional and local characteristics, socio-economic status etc. (Interview 8). Furthermore, there are data gaps concerning infection sites, conditions under which infections occur and how they are distributed by occupational groups, socio-economic status or social and leisure behaviour: “for 90 percent of Corona infections, we still do not know (…) were they occurred” (Interview 6). This gap is also related to the lack of social scientists in providing policy advice (see Schnell, 2021).

Considerable gaps also became apparent regarding the capacity-related data situation right at the beginning of the crisis. While these gaps were at least partially resolved with the establishment of the DIVI register, some parameters of health care facilities are not recorded nationwide. Specifically, this problem concerns the recording of staff coverage in hospitals, intensive care units, local health departments and nursing facilities.

#### Lack of Impact Data and Evaluative Knowledge

Another deficit has been identified in the provision and utilization of data to assess the consequential effects of the containment measures, as respective data is available only in fragmented and inadequate form. In this context, it is important to highlight data allowing for a more holistic pandemic assessment including indirect health effects, such as effects due to postponed treatment and surgeries, delayed diagnosis as well as lockdown-related domestic violence and abuse. The collection and utilization of such data are characterised as an essential requirement for data-based scientific policy advice and decision-making, as it is the only way to make an overall assessment of the pandemic and the effects of containment measures. Furthermore, a deficit of data on the effectiveness of certain pandemic management measures has been observed.

In addition, transnational data is conspicuously missing, which is particularly disadvantageous for border regions such as France, Poland and the Czech Republic. As reliable data on commuters is not available, counties face difficulties in assessing impacts of potential border closures, e.g., concerning health care facilities which rely on personnel from neighbouring countries. The lack of comparable European and international databases hinders a general overview of the pandemic in Europe. Especially regional and local decision-makers are thus forced to compile data on an ad-hoc basis, relying on random contacts and informal communication with colleagues in neighbouring countries.

### The Interface between Science, Public Administration and Politics

The interplay between policy-makers and scientific data providers as well as the use of data for the preparation and legitimation of decision-making processes has never been more pronounced than during the COVID-19 crisis (Interview 5). Consequently, the operational rationality of policy-makers has been and continues to be highly “data- and number-driven” (ibid.). Data collection and analysis are not the only central components of crisis management, keeping in mind that the overarching policy goal is to protect the health care system from overload. Policy-makers also see the necessity to justify their decisions by referring to scientific data and evidence, which is considered as a decisive factor for the society’s acceptance of measures and their willingness to comply.

At the federal level, institutionalized contacts with the scientific data providers of the departmental research institutions and with the scientific advisory boards of the ministries facilitated the integration of data in the decision-making process. However, political decisions on measures were largely based on external data providers, especially on virological and epidemiological expertise, but also on physical modelling and forecasts. While this already refers to the criticism on an ‘expertocracy’, further problems arose due to the media presence of some external knowledge providers, thus determining the political decision-making process. Due to the effects of public expectation, this had an impact on the scope of political action, sometimes hindering deliberative, data-based decision-making (Interview 10).

In accordance with the postulated need for ‘brokers’ and ‘translation’ (see above), major communication problems and uncertainties in the mutual expectations of scientific data providers and political-administrative decision-makers were observed, particularly during the initial phase of the COVID-19 crisis. Mutual understanding between both ‘worlds’ turned out to be difficult, as the “same language was not spoken” (Interview 11). Furthermore, an uncertain and volatile data situation combined with a dynamic development of the crisis complicates the weighing of data in the context of decision-making processes. In this situation, scientific statements characterised by contextualization and deliberative wording contrasted the demand of political-administrative decision-makers seeking to obtain unambiguous findings and precise recommendations. While scientific data providers have to emphasise the incertitude of forecasts and to provide complex explanations on the data context in order to meet the criteria of scientific working, this results in uncertainty for data users in politics and public administration. Due to such communication problems between scientific data providers and political-administrative decision-makers, it remains unclear whether and to what extent scientific data ultimately influenced and substantially determined the decision-making processes. Nevertheless, a clear learning curve and a mutual convergence between scientific data providers and political-administrative decision-makers were observed in the course of the COVID-19 crisis.

However, there are still deficits in the mutual understanding of respective roles between scientific data providers and political-administrative decision-makers. At various points, urgent calls were made for science as well as for politics to reflect on their respective roles. Thus, science has “advantages concerning its knowledge, but no democratic legitimacy” (Interview 8). Consequently, scientists were claimed to advertise and to convince political-administrative decision-makers in order to push forward their proposed solutions. In contrast, the latter were expected not to demand ready-made decisions from scientists, as their function consists of balancing and weighing normative values, taking into account different data-based interpretations and potentially conflicting societal interests when choosing one policy option while rejecting another one.

As mentioned above, the validity of knowledge concerning the COVID-19 pandemic is characterised by an unprecedented short-term, volatile and contested nature. This resulted in further problems, especially for political-administrative decision-makers: “it has never happened before, that every 6-8 hours the scientific recommendations changed completely” (Interview 10). Consequently, containment measures were sometimes adjusted in parallel. The discussion on closures of schools illustrates respective challenges: initially, policy-makers refrained from this measure citing scientific policy advice. As the scientific debate evolved and political perceptions changed, school closures were finally enacted irrespective of their initial rejection. The uncertainty and volatility of data and resulting policy recommendations, manifested in short-term, sometimes even fundamental changes of the current state of the academic debate, are characteristic features of this particular crisis, profoundly challenging consistent data-based decision-making.

In addition, various interviewees criticized the fact that political decision-makers at the federal and Länder levels failed to ensure sufficient transparency on the data and evidence they took into account to formulate containment measures. Given that the particular potential of data consists of ensuring acceptance of political measures, a lack of transparency about data relevant to decision-making and justifications for measures characterised by an insufficient database appears to be a particularly serious omission. This is mostly criticised by local decision-makers, as the above-mentioned aspects resulted in confusion and a lack of comprehension for rapidly changing measures. Thus, respective actors face increasing difficulties in convincing the local population of the meaningfulness of the measures and the need to comply with them.

A further criticism refers to the lack of transparency regarding the selection of data providers and experts as well as their respective mandates (Interviews 2 and 6). As a result, the answer to the question of which data was perceived by political decision-making too often depended on “who is closest to the German federal minister of health Jens Spahn” (Interview 6). Against this background, there is a call for the selection of data providers and experts to be made with greater transparency and more aligned with scientific criteria (recommendations, peer reviews, publications, etc.). Another problem is seen in the limited informational capacities and increasing information overload of politico-administrative actors, who face severe difficulties in “digesting” and processing all crisis-relevant information inputs adequately. Scientific advisors (particularly coming from departmental research institutions) complain about the “clogging of the pipeline” for data providers because policy-makers could not process the ever-growing loads of data and thus some serious bottlenecks of data transfer became apparent (Interview 8).

### Institutional Contexts of Data Utilisation

Data utilization largely depends on a supporting or hindering institutional infrastructure and administrative culture (see above). “Digital maturity” (cf. Coursey & Norris, 2008) and the “data culture” of administrative organizations (e.g., federal ministries), as well as the “data literacy” of involved actors are key variables to explain data (non-)utilisation in crisis-related policy advice and decision-making. It turned out that data utilization is frequently hindered by a missing awareness of the data available in-house and/or of its strategic relevance for organizational processes (Interview 11). Yet, even in cases of more extensive awareness, there are still challenges of data sharing across organizational boundaries. The extent to which data-based scientific policy advice can actually reach policy-makers largely depends on the extent and quality of (digital) data sharing. In this context, significant deficits and bottlenecks had become evident even before the COVID-19 crisis. “Departmental silos” (see Bundesregierung, 2021: 59) restrain the access of various departments to shared databases and the cross-departmental re-use of data. Data tends to be ‘hoarded’ in one’s own organisation instead of being shared with other administrative units.

The extent and intensity of cross-organisational data exchange largely depends on the respective data tradition of the organisation and the professional backgrounds. Administrative departments with predominantly legal professional backgrounds and/or little experiences in data-driven processes have proven to be less cooperative and open to data-sharing. In contrast, those departments with a long-standing practical experience in dealing professionally with larger volumes of data on a daily basis and/or professional backgrounds rather dominated by data and social scientists are more open to data-sharing and less inclined to data “hoarding”.

Furthermore, the fostering of competencies regarding the legal context of data utilization and data privacy rules is necessary. Even in cases of a predominant data-based operational rationality, there is widespread uncertainty on the scope for action regarding the regulatory conditions of data utilization and data sharing because it is unclear what is legally permitted and what is prohibited. The certainty of action with regard to the question of “whether and how to use data” (Interview 11) and an administrative culture anchoring data-oriented policy-making are still extremely limited in public administrations. These application hurdles of data protection rules, rather than the regulations as such, are the main source of problems with data utilization in public administrations.

Moreover, there is a lack of machine-readable provision of raw data, which leads to deficiencies in demand-responsiveness in data management. Due to this limitation, it has only been possible to use data for its intended purpose, whereas the ‘re-use’ of data in different contexts and for different problems has only occurred to a limited extent. Furthermore, administrations are lacking the necessary institutional arrangements and organizational structures for data-driven processes, both for their standard operations and for crisis management. Consequently, important data compilations and checks are often carried out manually, which causes additional personnel expenses and does not make work easier, which also represents an “add on” to normal working procedures. Thus, the growing need in politics and administration, especially in times of crisis, to obtain data across levels and organizations and to feed it into decision-making processes cannot be satisfied due to the lack of functioning process structures.

## Discussion: The Role of Interfaces and Contexts for Data-Based Scientific Policy Advice

Our analysis has shown that the strong data and number-driven orientation of policy decisions in the wake of the COVID-19 crisis initially suggested an approach to the technocratic model of policy advice (Böcher et al., 2021; Florack et al., 2021). This becomes apparent inter alia in decision-makers’ reference to data as their “staff of life”. Furthermore, our interviewees perceived the volatility and uncertainty of databases as serious limitations of political action in the crisis. In addition, the partly fundamental ad hoc changes of direction in crisis responses (e. g. keeping schools open vs. school closures) appear to reveal a predominantly technocratic understanding of advice (Kuhlmann & Franzke, 2021). Political decision-makers undertook these radical shifts in direction (sometimes overnight) with the justification that they were simply forced to execute the guidelines of “the science”. Against this background, at first glance, the assumption made above could be accepted, according to which the COVID-19 pandemic had played into the hands of a technocratic model of policy advice and that the instructions of science were only carried out by politico-administrative decision-makers.

On closer inspection, however, the assumption of a dominant technocratic model, in which experts sit in the “driver seat” and politicians only “in the back seat” of pandemic policy, cannot be maintained. Our interviews have made clear that, at least with regard to the federal level, during the crisis there has been a partial convergence in the perceptions and understandings of politico-administrative decision-makers on the one hand and scientific advisors on the other. Iterative processes of mutual learning, translation and transfer took place in which the cognitive and institutional boundaries between the two “worlds” of scientific advice and decision-making became blurred and the respective rationale for action partially converged. Based on our analytical framework, these processes can be regarded as “boundary work” and “boundary spanning”. This corresponds more to the pragmatic model of policy advice, which is characterized by complex interdependencies and close interactions between politicians and advisors.

This phenomenon is not only expressed by learning curves and mutual convergence of perceptions, but also by problematic overlaps between the two spheres. The latter resulted in an increasing politicization of scientific advisors, when in policy makers’ narratives value-based considerations and the political appreciation of different interests were replaced by way of referring to an allegedly scientifically determined lack of alternatives. The resulting overall deficit in role transparency increased rather than shrunk over the course of the crisis (see Beck & Nardmann, 2021). The pragmatic model of policy advice, which is characterized by boundary spanning, mutual adaptation, learning and transfer, has thus also revealed a number of problematic consequences and dysfunctions. This in turn has brought up demands for a clearer separation of roles, i.e. for shifting to a (traditional) dualistic model of policy advice, which, as empirical research shows, is to be regarded however as rather unrealistic.

While our empirical results can thus be interpreted primarily as confirming the pragmatic model of policy advice, there is also some evidence which supports the assumption of a decisionist model. This affects decisions on some measures (e. g. regarding lockdowns) for which scientific advice from medicine and virology was obtained. However, the evidence-base for these measures to effectively combatting a pandemic was rather uncertain, volatile and controversial, respective policy choices varied across countries (e.g. Swedish vs. French approach; see Kuhlmann et al., 2021; Böcher et al., 2021: 359) and the advice obtained was mainly restricted to virological and medical aspects of the pandemic. Nevertheless, decision-makers justified their choices by referring to “the science” (and “the numbers”) and thus used this narrative to legitimize severe policy choices which possibly had been taken in advance^7^ and were in need of an undisputable scientific backing (for a critical assessment see inter alia Wiesing et al., 2021; Hirschi, 2021). Multi-disciplinary and controversial debates on various options of crisis mitigation, in which different disciplinary approaches could have been subjected to a more balanced and holistic assessment of the pandemic, were clearly neglected.

Our findings regarding the transparency deficits and lack of disciplinary breadth in the selection of experts by executive politics further point to a decisionist structure of the advisory system in which knowledge is primarily used for symbolic-legitimizing purposes (see Blum et al., 2021: 254). The reference to scientific evidence was de facto a vehicle for decision-makers to narrow down the discourse (and thus the possible policy options) to a monodisciplinary “biomedicine-centric” demonstration “with marginalized input from non-biomedical disciplines” (Lohse & Canali, 2021: 20). Through the strategic use of technocratic arguments, it was possible to make respective decisions unassailable, albeit at the expense of more creative policy options which could have been inspired by more than just one or few disciplines. At the same time, the strategic use of technocratic arguments naturally had the important function of increasing citizens’ acceptance to comply with the measures and to increase the public willingness to follow the rules. In this logic, it was possible for decision-makers to frame their choices as unavoidable since, allegedly, they were only the result of what “the science” prescribed. However, this form of strategic knowledge use (cf. Johnson et al., 2009) and the established narrative offered the opportunity to externalize the responsibility for policy choices, including the potentially negative consequences and collateral damages (“blame shifting”, see Mortensen, 2016: 632ff).

In summary, with regard to our analytical framework, the assumption of a predominantly technocratic model of policy advice, which is promoted by well-established structures and functioning processes of data-driven government, cannot be confirmed. Instead, the reality largely corresponds to the pragmatic model, in parts also to the decisionist model, albeit with sometimes highly dysfunctional characteristics. In many cases, the actors make use of technocratic arguments and strategic use of knowledge in order to legitimize their decisions, promote acceptance of measures and to shift responsibility for the potentially negative consequences of drastic measures.

The institutional context factors, of which above all the structural, cultural and process-related aspects of data management were examined here, tend to have an inhibiting and limiting effect on data-based scientific policy advice. This applies in particular to the boundary-spanning mechanisms in the pragmatic model. Due to the deficits in data quality, data sharing and data accessibility identified here, as well as an insufficiently developed administrative data culture and data literacy, there are restrictions in the data flow between scientific advisors and politico-administrative decision-makers as well as within the administration and between different administrative sectors. Necessary structures of vertical coordination and multi-level data management (e. g. in the reporting system) were hardly functioning, which in turn had negative impacts on the overall effectiveness of data-based scientific policy advice and thus on crisis governance as a whole.

## Conclusions

The study shows that data-based scientific policy advice has played a key role in pandemic management and decision-making. However, several deficits and problems with regard to the availability, quality, accessibility, divisibility and usability of data were identified, posing considerable challenges to data producers and users. For the two key dimensions of the analysis (interfaces and contexts), the following key findings can be summarized:

The interface between scientific advisors and policy-makers has been marked by multifaceted role conflicts, ambiguities, and tensions which have obviously been more pronounced in the pandemic. It has become apparent that policy-makers were literally dependent on data, not only to take better informed and more evidence-based decisions, yet to enhance the legitimacy of unprecedented and far-reaching policy decisions and thus to foster citizens’ compliance with those. Against this background, it turned out to be a major problem, that especially in the early phase of the pandemic, considerable communication problems and uncertainties in the mutual expectations of scientific data providers and politico-administrative actors became apparent. The awareness and mutual understanding of the specific roles of actors involved in advisory process and their role delimitation was considered inadequate. It was in particular criticized that policy-makers tended to shift blame to scientists and thus to escape political accountability by way of advocating a technocratic model of policy advice according to which they would find themselves as determined by science and forced to merely execute scientists’ directives when taking political decisions on pandemic containment measures.

Against this background, there seems to be a need to have a more clear-cut separation between scientific advisors and policy-makers, with the former limiting themselves to supporting the preparation of political decisions and enriching them with data. The latter is expected, by contrast, to refrain from any demands of receiving ready-made political decisions from scientists, yet to accept and fulfil their role of being responsible and accountable for balancing and evaluating potentially controversial knowledge sources, data interpretations and conflicting values on a normative basis. However, policy-makers are of course recommended to base their decisions on available data and knowledge sources more systematically when balancing conflicting goals, values, opinions and recommendations before - ultimately - taking a well-grounded normative decision which they will be held accountable for.

Furthermore, the findings have revealed the institutional and cultural context of data generation, transfer and utilization being a salient explanation for deficits in data-based scientific policy-advice and pandemic management. Inter alia, glaring problems of interoperability were observed when it comes to exchanging data between laboratories, municipal, *Länder* and federal health authorities as well as significant digitalization backlogs at all levels of government, yet most conspicuously at the municipal level. Another challenge of data-based scientific policy advice is to bundle, process and make accessible the amount and variety of existing data in such a way that various decision-makers can access and (re)-use these data for different purposes. However, such efforts have often failed so far because of “silo thinking” and “in-house data hoarding” and a limited willingness to share and exchange data across administrative organizations. Additionally, the lack of an interdisciplinary profile, particularly regarding ministerial bureaucrats’ qualifications, hinders the development of a “culture of open data” and for data to become a strategic resource of decision-making and controlling. This has in particular become apparent in those authorities with a predominant legal professional background.

Overall, the study shows that data-based policy advice and decision-making are not only to be improved by way of producing more data and enhancing its quality, connectivity and divisibility, which would be a rather technocratic understanding of policy advice. Yet, more emphasis is to be put on the incentive structures, motivations, as well as the “will and skill” of advisors on the one hand and decision-makers on the other. For future advisory processes the key challenge must be addressed in order to better reconcile the rationality of facts, information, and evidence with the rationality of power, compromise, and legitimacy to ultimately promote more evidence-based decisions in times of crisis and beyond.

## Appendix

**Table 1:** Expert interviews carried out in the project (April/Mai 2021)

No.	Institution	Group Assignment	Hierarchy Level
1	Robert-Koch-Institute	Science/intern	Head of department
2	Federal Chancellery	Politics/administration (federal level)	Unit level
3	Head of County Administration Office	Politics/administration (local level)	Head of County Administration
4	Foundation „Neue Verantwortung“	Non-governmental organizations/foundations	Project management
5	State Chancellery	Politics/administration (Länder level)	Team leader
6	Hasso-Plattner-Institute	Science/extern	Professor
7	Leibniz Institute for Economic Research	Science/extern	Management level
8	Federal Institute for Population Research	Science/intern	Management level
9	Federal Office for Civil Protection and Disaster Aid	Science/intern	Head of department
10	Federal Ministry	Politics/administration (federal level)	Head of department
11	Federal Ministry	Politics/administration (federal level)	Head of department/Head of Unit
12	Federal Commissioner for Data Protection and Freedom of Information	Politics/administration (federal level)	Head of department
13	German Association of Cities	Politics/administration (local level)	Representative
14	Fraunhofer-Institute	Science/extern	Management level
15	Open Knowledge Foundation	Non-governmental organizations/foundations	Strategist
16	Organisation for Economic Co-operation and Development (OECD)	Science/extern	Unit/Project management

Source: Own compilation

## Footnotes

1 Our article is based on the findings of a research project funded by the German Federal Ministry of Education and Research (BMBF) which forms part of the research consortium “Research and Advice on Crisis Management of the COVID-19 Pandemic”.

2 The federal emergency brake automatically applied when – within a defined period of time - a certain number of COVID-19 cases (positive PCR tests) is counted in a territory, legally binding national standards and indicators for pandemic containment were stipulated, thus replacing local assessments in this regard.

3 Due to the Länder and municipalities’ competence in issuing pandemic regulations, there has been a high degree of variety regarding the regulatory details of pandemic containment and mitigation measures, such as social distancing, “stay at home rules”, wearing of face masks, testing and tracing, protection of the elderly etc. Yet, some of these differences have become blurred over the course of the crisis and, particularly in phases of crisis aggravation. As a result, there was a general trend towards regulatory convergence across the Länder, triggered by intensified intergovernmental coordination and even centralization of decision-making during the crisis (see Kuhlmann & Franzke, 2021). For a more comprehensive and detailed analysis of the German regulatory landscape in the 16 Länder during the pandemic and the explanatory factors of intra-federal variation see inter alia: Behnke and Person (2021) on variation of protection measures in the German Länder, Glogowsky et al. (2021) on effectiveness of social distancing policies and Sack et al. (2021) on German economic policy in the Covid-19 pandemic.

4 Taking the particularities of the COVID-19 crisis into account, to these “two worlds” the rationality of administrative practice, implementation and enforcement of decisions would have to be added (as a “third world”).

5 The Robert Koch-Institute is a departmental research institute of the federal ministry of health directly sub-ordinated to the latter.

6 The DIVI register is based on a cooperation between the German Interdisciplinary Association for Intensive and Emergency Medicine (DIVI) and the Robert Koch Institute. It was introduced during the first months of the COVID-19 crisis in 2020 and monitors data on the capacity situation of Intensive Care Units (ICUs) in Germany.

7 This assumption was put forward in particular with regard to the 7th ad hoc statement of the National Academy of Science Leopoldina which recommended a strict lockdown end of 2020 (see Hirschi, 2021; Leopoldina, 2020).
